# Analysis of Cancer Stem Cell Markers in Various Histological Subtypes of Adrenocortical Cancer

**DOI:** 10.3390/cimb46120825

**Published:** 2024-12-05

**Authors:** Nano Pachuashvili, Asya Bastrich, Erika Porubayeva, Alina Elfimova, Alexander Tertychnyy, Dmitry Beltsevich, Evgeniya Kogan, Igor Reshetov, Ekaterina Troshina, Natalia Tarbaeva, Natalia Mokrysheva, Liliya Urusova

**Affiliations:** 1 Department of Fundamental Pathology, Endocrinology Research Centre, 117036 Moscow, Russia; 2 Institute of Clinical Morphology and Digital Pathology, I.M. Sechenov First Moscow State Medical University, 119991 Moscow, Russia; 3 Faculty of Fundamental Medicine, Lomonosov Moscow State University, 117192 Moscow, Russia

**Keywords:** cancer stem cells, adrenocortical cancer, markers of cancer stem cells, markers of multipotent stem cells

## Abstract

Adrenocortical cancer (ACC) is a rare malignant neoplasm originating from the adrenal cortex, presenting limited therapeutic options. An avenue for improving therapeutic efficacy may involve a deeper understanding of the role of adrenocortical stem/progenitor cells in the pathogenesis of this disease. Although existing data suggest stem/progenitor characteristics in certain cell populations within ACC, the challenge remains to identify adrenocortical stem cell markers directly involved in its carcinogenesis. In our study, we aimed to identify multipotency markers such as LGR5 and CD90 in various ACC types to confirm their presence and localization. The study included tumor tissue samples from 13 patients with ACC treated at the Endocrinology Research Centre (Moscow, Russia) between 2005 and 2023. We conducted immunohistochemical analyses to identify the aforementioned markers and examined the association between their expression and clinico-morphological parameters. Our pilot study results demonstrate the presence of LGR5- and CD90-positive tumor cells in all samples. Despite the small sample size, we observed statistically significant differences in disease-free survival based on the number of CD90-positive cells. These findings suggest a potential diagnostic, prognostic, and predictive value of cancer stem cell markers, underscoring the need for their further analysis in a larger cohort of patients with ACC.

## 1. Introduction

The adrenal cortex has a dynamic cellular structure capable of self-renewal throughout life: aging cells are replaced by new ones, and metabolism, along with the width of functional zones, is regulated to meet the physiological need for steroids or to respond adequately to external pharmacological stimuli. Renewal of the adrenal cortex occurs both as a result of damage and under normal homeostatic conditions. At the same time, the entire cortex is replaced every few months [1].

In the adult adrenal gland, various populations of cells are described that are located in the adrenal capsule and cortex, involved in maintaining homeostasis of the adrenal cortex [2,3,4,5,6]. These capsule and cortical populations interact via reciprocal signaling networks, using mechanisms that are not well understood, coordinating centripetal proliferation and differentiation in response to paracrine and endocrine signals. However, we still do not have a clear understanding of how the niche of somatic stem cells in the adrenal cortex functions, i.e., the biological properties of these cells, their division rate, and migration ability, are controlled.

According to modern theories of carcinogenesis, cancer develops as a result of the accumulation of oncogenic mutations in the DNA molecule. The risk of DNA damage is particularly high during cell division and differentiation. Stem cells are the only cells that are able to preserve DNA from the zygote stage throughout the life of the organism and that are able to divide, self-renew, and differentiate throughout their existence. Both of these facts strongly suggest that, in most cases, the occurrence of cancer is associated with the oncotransformation of somatic stem cells into cancer stem cells [7,8]. As a consequence, organs with high regenerative potential, such as the intestine, respiratory tract, liver, and pancreas, have a high incidence of cancer [9].

The transcription profile of cancer stem cells (CSCs) has much in common with the transcription profile of normal somatic stem cells; they share similar cellular characteristics, including surface/intracellular markers and intercellular signaling pathways. As a consequence, CSCs show striking similarities with normal somatic stem cells in terms of long-term proliferation and an apoptosis inhibition program [10]. Pathways involved in the self-renewal of normal somatic stem cells and the maintenance of stemness contribute to the proliferation and invasion of CSCs [11].

Currently, various CSC markers have been characterized [12,13], many of which are multipotency markers [14]. Some of the most well-characterized CSC markers are involved in signaling pathways that are crucial in the regulation of the somatic stem cell pool of organs.

**Figure 1:**
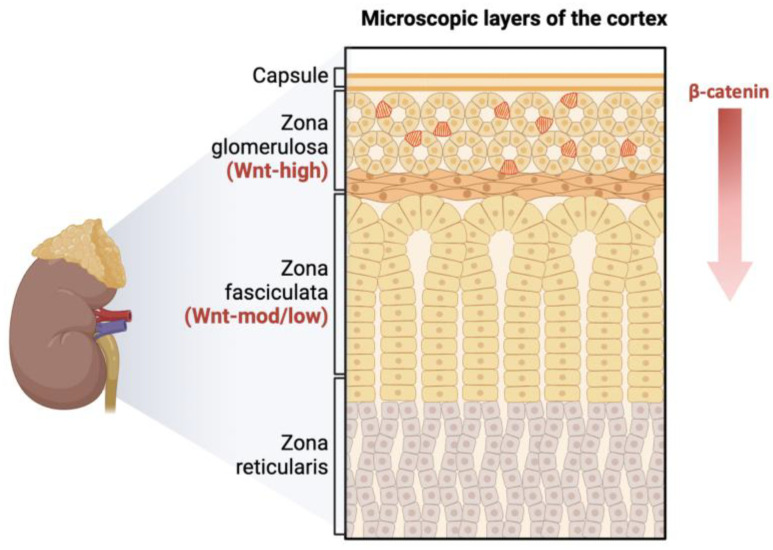
The adrenal cortex is maintained through centripetal differentiation. It is composed of concentric zones that are histologically and functionally distinct. *Wnt/β-catenin* signaling exists in a centripetal gradient within the adrenal cortex. The red text (Wnt-high and Wnt-mod/low) indicates the activity levels of the Wnt/β-catenin signaling pathway in different areas of the adrenal cortex. The adrenal zona glomerulosa is marked by high *Wnt* activity. The zona fasciculata is marked by moderate/low *Wnt* activity. As zona glomerulosa cells differentiate, β-catenin is inhibited, allowing for transdifferentiation from zona glomerulosa to zona fasciculata and subsequent corticosteroid production.

LGR receptors and their RSPO ligands are essential components in the *Wnt/β-catenin* signaling (Figure 1). The Wnt-potentiating *RSPO/LGR* signaling module has also been found to be aberrantly activated in a variety of cancers. LGR5, a marker of intestinal stem cells, promotes colorectal cancer cell survival in mice and was found to be overexpressed in 64% of one-patient cohorts [15,16,17]. Upregulation of LGR5 has also been noted in several ovarian, breast, and hepatocellular cancers [18]. In addition, a recent study from Al-Bedhawi showed that a few specific types of cells within the rat adrenal capsule expressed a high level of CD90 [19]. CD90, as a cell surface antigen, is considered one of the mesenchymal multipotent stem cell markers [20].

The objective of this study was to confirm the presence and localization of the aforementioned markers of multipotency in the normal human adrenal gland tissue, as well as to identify and characterize them in various types of ACC. The obtained results demonstrate the presence of LGR5- and CD90-positive tumor cells in all samples. Our results also suggest that there are at least two populations of stem cells in the human adrenal cortex, which are spatially separated and supported by different signaling systems. We also have found that LGR5+ cells and β-catenin activity exist in the zona reticularis despite the fact that in this part of the adrenal cortex, β-catenin should be inhibited by *PKA* signaling [18].

## 2. Materials and Methods

### 2.1. Patients and Samples

The study included tumor tissue samples from patients with adrenocortical cancer who were treated at the Endocrinology Research Centre. All patients underwent adrenalectomy in the period from 2005 to 2023. Patients under the age of 18 at the time of surgery were excluded from the study. Importantly, none of the patients included in the study had received any neoadjuvant therapy prior to surgery. A total of 13 cases of adrenal tumors were selected for the study. All tumor tissue samples were verified in accordance with the 2022 WHO classification of adrenal cortical tumors. The study included 3 cases of conventional subtypes of adrenocortical cancer, 3 oncocytic, 3 myxoid, and 4 cases of mixed tumor (conventional and oncocytic subtypes).

The study also included 2 samples of normal adrenal tissue obtained from surgical material from patients who underwent surgery for renal tumors. Tissue samples were taken at a distance from the tumor, and the presence of pathological formations in the material was completely ruled out.

### 2.2. Immunohistochemistry Imaging

Tumor tissue samples obtained during surgical treatment were fixed in 10% buffered formalin, processed in the histological wiring system of Leica ASP200 (Leica Biosystems, Wetzlar, Germany) and embedded in paraffin. Further, paraffin sections with a thickness of 3 µm were made from the paraffin-embedded tumor tissue samples on the microtome and applied to slides treated with poly(L-lysine).

Immunohistochemical (IHC) analysis of tumor tissue sections was carried out using a standard technique with a peroxidase detection system employing DAB on an automatic Leica BOND III IHC staining system (Leica Biosystems, Wetzlar, Germany). The antibodies used in this study were anti-CD90 rabbit monoclonal antibody, ab133350 (Abcam, Cambridge, MA, USA); anti-LGR5 rabbit polyclonal antibody, ab219107 (Abcam, Cambridge, MA, USA); and Ki-67 antibody, MIB-1 (Dako, Glostrup, Denmark). All histological slides were scanned using a Leica Aperio AT2 system at 20× magnification for further analysis.

In this study, cell detection and quantification were performed using the open-source software QuPath (version: 0.5.1). The counting of CD90-positive cells was performed in five fields of view, each measuring 0.05 mm² (total area of 0.25 mm²) separated into areas for the tumor parenchyma and the subcapsular region. The most representative fields of view were selected. A semi-quantitative approach was used to assess LGR5 expression. Samples were scored on a scale from 0 to 3 in ascending order: 0 for negative samples or those with less than 10% stained tumor tissue; 1 for samples with 10 to 39% stained tumor tissue; 2 for samples with 40 to 79% stained tumor tissue; and 3 for tumors with 80% or more stained tumor tissue. Scores were determined independently by three pathologists. Clinicopathological information was concealed from the observers.

The Ki-67 percentage score is defined as the percentage of positively stained tumor cells among the total number of malignant cells assessed in 10 high-power fields at ×400 magnification in areas of the tumor with the highest activity («hot spots»).

### 2.3. Statistical Analysis

Statistical analysis was conducted using Python 3.9, utilizing libraries such as pandas, numpy, sklearn, scipy, and lifelines.

Descriptive statistics for quantitative features are presented as medians along with first and third quartiles, while qualitative features are reported in terms of absolute and relative frequencies. The comparison of three or more independent groups on a quantitative basis was performed using the Kruskal–Wallis criterion, followed by post hoc analysis. Correlation analysis was conducted using Spearman’s method.

Survival analysis was carried out using Kaplan–Meier analysis. A log-rank test was employed to compare the survival of two groups. Cox regression analysis was used to construct prognostic survival models. The critical level of statistical significance was set at 0.05. For multiple comparisons, the level of statistical significance was corrected using the Bonferroni correction.

## 3. Results

The study included 13 tumor samples from 13 patients with ACC, comprising 12 females with a median age of 54 years (Q1–Q3: 37–59) and 1 male patient aged 35 years. The clinical and morphological characteristics of the study group are presented in Table 1.

### 3.1. Analysis of CD90 Marker Expression

A positive immunohistochemical reaction with the CD90 marker was detected in all studied ACC samples. The number of CD90-positive cells under the capsule and in the tumor parenchyma was 26 (Q1–Q3: 10–91) and 28 (Q1–Q3: 13–207) cells/mm², respectively.

**Figure 2:**
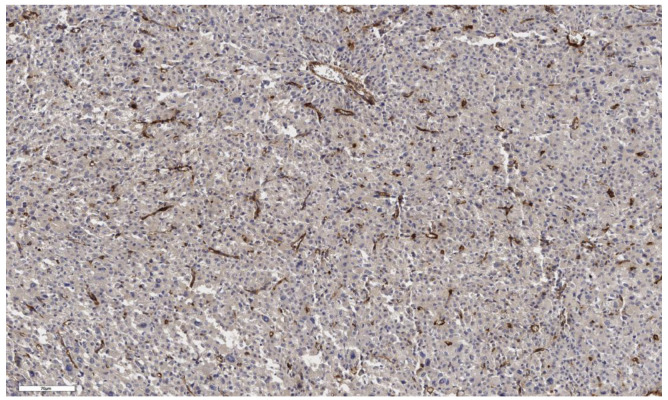
Conventional subtype of ACC. The parenchyma of the tumor. IHC reaction with CD90 marker, ×100. Scale bar = 70 μm.

**Figure 3:**
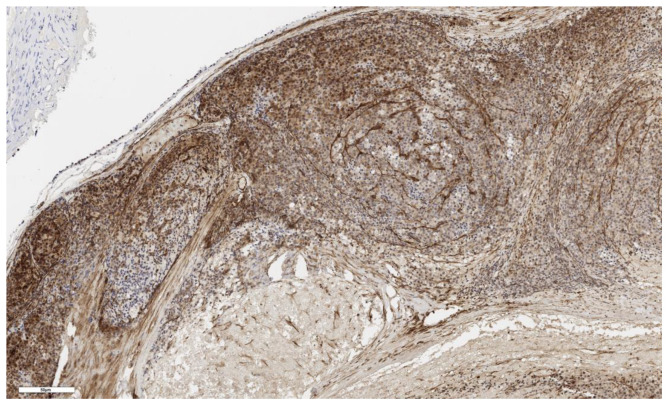
Conventional subtype of ACC. CD90 positive cells under the tumor capsule, ×60. Scale bar = 50 μm.

**Figure 4:**
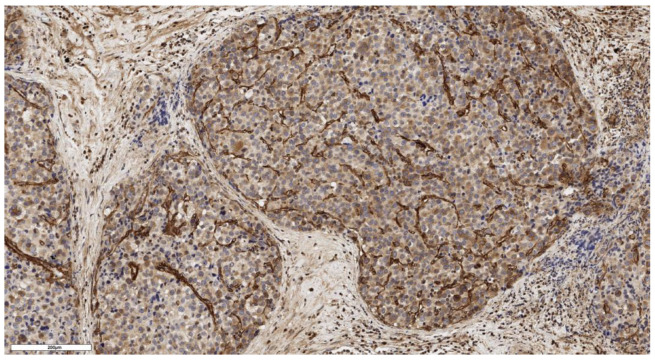
Mixed subtype of ACC. The parenchyma of the tumor. IHC reaction with CD90 marker, ×150. Scale bar = 200 μm.

**Figure 5:**
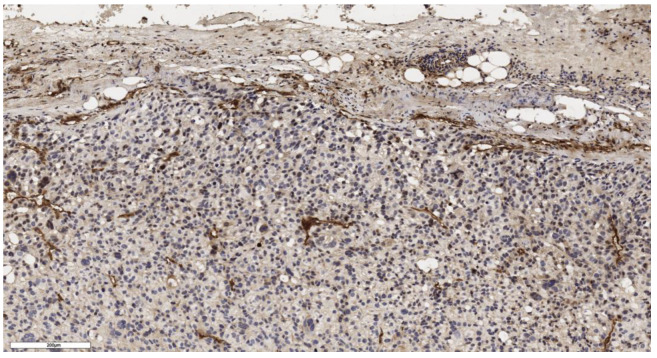
Mixed subtype of ACC. CD90 positive cells under the tumor capsule, ×100. Scale bar = 200 μm.

An analysis of the association between CD90 expression and the clinicopathological data of patients was conducted (Table 2). The number of CD90+ cells in the tumor parenchyma was relatively higher in samples of conventional and mixed histological subtypes compared to other histological subtypes of ACC (Figure 2, Figure 3, Figure 4 and Figure 5); however, these differences did not reach statistical significance (*p* = 0.161). No significant differences in CD90 expression beneath the tumor capsule were found across histological subtypes either.

**Figure 6:**
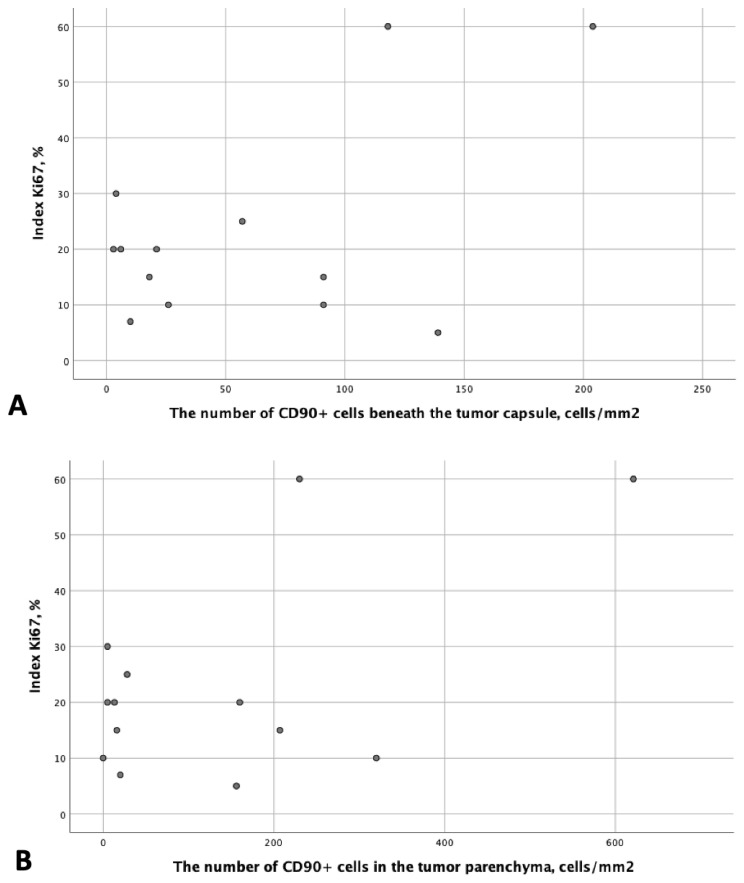
The results of the correlation analysis between the Ki67 proliferative index and the number of CD90+ cells beneath the tumor capsule (**A**) and in the tumor parenchyma (**B**).

A correlation analysis was conducted between the Ki67 proliferative index and the number of CD90+ cells beneath the tumor capsule and in the tumor parenchyma (Figure 6). However, no statistically significant differences were found (*p* = 0.762 and *p* = 0.353, respectively).

An analysis of the dependence of the number of CD90+ cells beneath the tumor capsule and in the tumor parenchyma on the disease stage according to the ENSAT classification was performed, and no statistically significant differences were identified in the correlation analysis (*p* = 0.707 and *p* = 0.633, respectively).

The analysis of the number of CD90+ cells beneath the tumor capsule and in the tumor parenchyma was performed depending on the size of the primary tumor (T stage); however, no significant differences were obtained (*p* = 0.687 and *p* = 0.73, respectively). Analysis of the number of CD90+ cells beneath the tumor capsule and in the tumor parenchyma, depending on the presence of metastases in regional lymph nodes (N stage), also revealed no significant differences (*p* = 0.887 and *p* = 0.811, respectively). No significant differences in the significance of the presence of distant metastases (M stage) on the number of CD90+ cells beneath the tumor capsule and in the tumor parenchyma were found (*p* = 0.19 and *p* = 0.28, respectively).

### 3.2. Analysis of LGR5 Marker Expression

**Figure 7:**
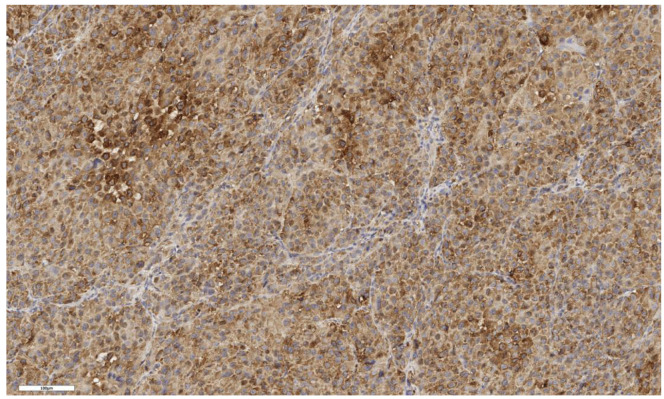
Conventional subtype of ACC. IHC reaction with Lgr5 marker, ×150. Scale bar = 100 μm.

**Figure 8:**
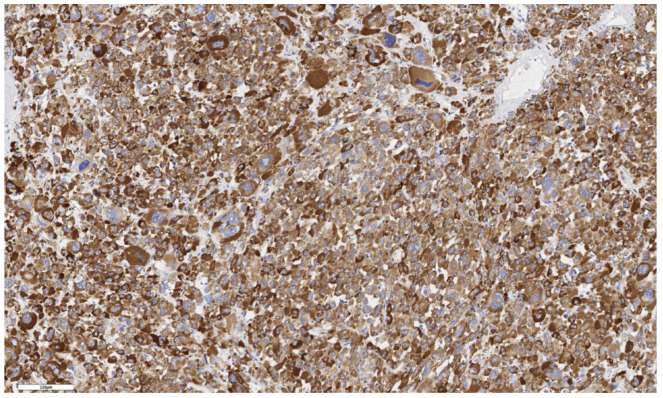
Oncocytic subtype of ACC. The parenchyma of the tumor. IHC reaction with Lgr5 marker, ×150. Scale bar = 120 μm.

An LGR5 expression scored as 1 was observed in six cases (46%), as 2 in four cases (31%), and as 3 in three cases (23%). In eight cases (62%), LGR5 expression was focal, while in five cases (38%) it was diffuse (Figure 7 and Figure 8). An analysis of LGR5 expression based on the histological subtype of ACC (Table 3) revealed no significant differences (*p* = 0.199).

In the correlation analysis of the dependence of LGR5 expression in cells on the disease stage according to the ENSAT classification, no statistically significant differences were identified either (*p* = 0.199).

The analysis of LGR5 expression in cells was performed depending on the size of the primary tumor (T stage); however, no significant differences were obtained (*p* = 0.267). Analysis of LGR5 expression in cells depending on the presence of metastases in regional lymph nodes (N stage) also revealed no significant differences (*p* = 0.31). There were no significant differences in the significance of the presence of distant metastases (M stage) and LGR5 expression (*p* = 0.253, respectively).

### 3.3. Analysis of Patient Survival Depending on the Expression of Stem Markers

**Figure 9:**
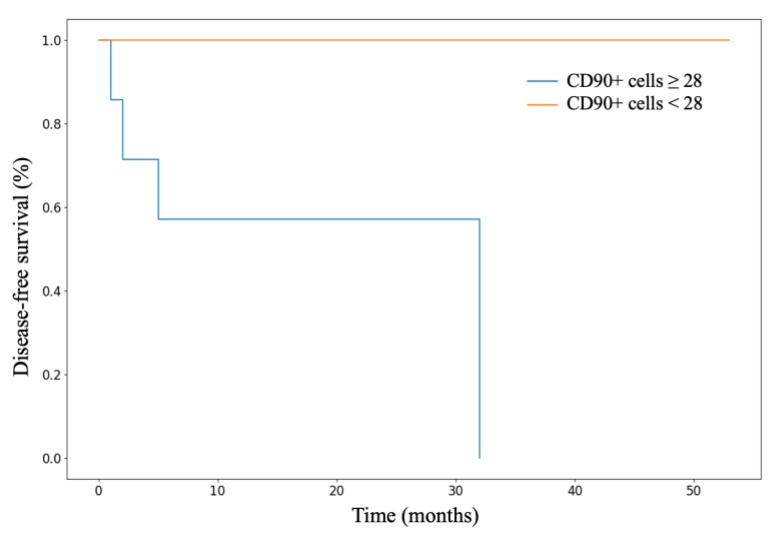
The Kaplan–Meier curves for disease-free survival of patients with ACC depending on the number of CD90+ cells in the tumor parenchyma.

We conducted an analysis of patient survival based on characteristics of stem marker expression. Statistically significant differences were found in the disease-free survival (DFS) rates of patients with ACC depending on the number of CD90+ cells in the tumor parenchyma (*p* = 0.042). Kaplan–Meier survival curves are presented in Figure 9. Patients with a number of CD90+ cells below the median value demonstrated better DFS compared to those with a number above the median (*p* = 0.042). 

### 3.4. Analysis of Stem Marker Expression in Normal Adrenal Tissue

**Figure 10:**
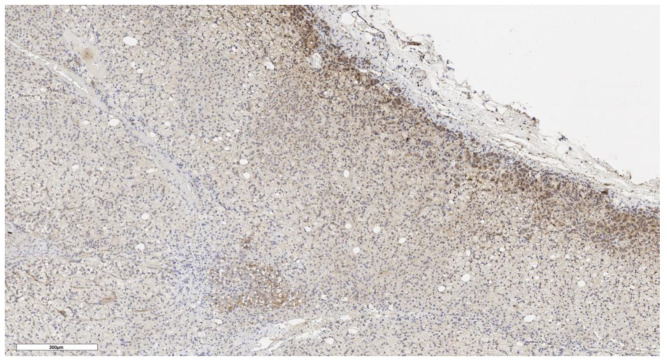
CD90 expression in normal adrenal tissue, ×100. Scale bar = 300 μm.

**Figure 11:**
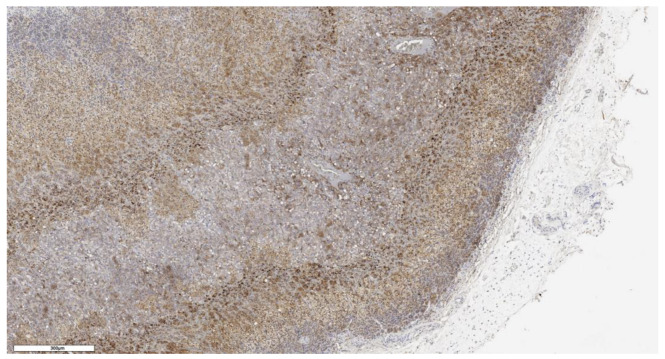
Lgr5 expression in normal adrenal tissue, ×100. Scale bar = 300 μm.

In an immunohistochemical study of normal adrenal gland tissue using CD90 and LGR5 markers, a substantial number of cells stained positive. Notably, cells that stained positively for CD90 were located beneath the organ’s capsule (Figure 10); no positive cells were detected in other zones of the adrenal gland. Conversely, cells expressing LGR5 were predominantly found in the zona reticularis and beneath the organ’s capsule (Figure 11). This distinct localization suggests specific roles for these markers in different regions of the adrenal gland.

## 4. Discussion

ACC is a malignant neoplasm originating from cells of the adrenal cortex, with an incidence of 0.7–2 cases per 1 million population per year [21]. Although radical tumor removal can potentially cure patients in some cases, the prognosis for a significant proportion remains unfavorable: the median overall survival (OS) is about 15 months [22]. Currently, therapeutic options for these patients are extremely limited. The use of the adrenolytic drug mitotane, in combination with other chemotherapeutic agents, has not demonstrated a significant improvement in survival for most patients, and targeted immunotherapy is only effective in limited cases [23]. The development of new, more effective, and safer methods of ACC treatment is now as relevant as ever.

Recent studies have demonstrated that CSCs contribute to the phenotypic heterogeneity of primary tumors, playing a crucial role in the initiation, growth, and metastasis of malignant neoplasms [24]. Furthermore, CSCs are significant in promoting immune evasion, thereby diminishing the effectiveness of cancer treatments. One avenue for enhancing therapeutic efficacy in treating patients with ACC may involve a deeper understanding of the role of adrenocortical stem/progenitor cells in the pathogenic mechanisms of disease onset and progression. Although existing data indicate the presence of stem and progenitor characteristics in certain ACC cell populations, the challenge of identifying adrenocortical stem cell markers directly involved in the carcinogenesis of this disease remains unmet.

A number of markers have been proposed to identify and characterize subpopulations of cells with CSC properties [24]. It is important to recognize that currently, there is no universal, standardized marker for CSCs that is both highly sensitive and specific. Previous studies have shown that various mechanisms are involved in the development and maintenance of CSC populations, and even within a single tumor, different types of CSCs with distinct marker expressions and properties can coexist. CD90 (also known as Thy-1) is a glycoprotein expressed on the surface of both tumor and immune cells. The phenotype of CD90-positive CSCs has been detailed in gastric cancer cell lines [25]. In this study, it is demonstrated that CD90-positive cells are associated with high tumor cell proliferation through cell cycle progression in some gastric cancer cultures. The CD90 surface antigen is now considered one of the markers of mesenchymal multipotent stem cells.

Leucine-rich repeat-containing G-protein-coupled receptor 5 (LGR5), also known as G-protein-coupled receptor 49 (GPR49), is a well-studied stem cell marker expressed in several organs, including the stomach, small intestine, colon, and liver [26]. It has been reported that selective ablation of LGR5-positive CSCs leads to tumor regression, and exposure to colon LGR5-positive CSCs enhances the effect of chemotherapy [27]. The presence and role of these markers in the adrenal gland have been previously described but not investigated in ACC until now.

To our knowledge, this is the first time the presence and localization of the multipotent stem cell markers CD90 and LGR5 in different subtypes of ACC have been confirmed. In particular, unlike previous studies, we also detected the expression of the LGR5 marker in normal adrenal cortex tissue not only in the zona glomerulosa but also in the zona reticularis. Immunohistochemical expression of the studied markers was detected in all 13 cases of ACC. Therefore, the IHC of samples from normal adrenal cortex tissue suggests that there are not one but at least two populations of stem cells in the adrenal cortex, which are spatially separated and supported by different signaling systems. Identification of stem cell subpopulations can contribute to the development of a cell model of the adrenal cortex, as well as a self-renewing cell model of ACC. The main common difficulty here is still the lack of a clear understanding of the critical signaling pathways involved in maintaining and differentiating stem cells. Studies on organs where somatic stem cell niches have been identified confirm that culture media effective for somatic stem cells [28] also work with minor changes for cancer stem cells [29]. Cells are provided with a culture medium consisting of a growth factor cocktail to trigger a regenerative response in the stem cells of the pertinent epithelium according to niche factor requirements. For example, key components for preparing human colon organoid culture medium include activators of Wnt signaling, ligands of tyrosine receptor kinases, and inhibitors of transforming growth factor-β/bone morphogenetic protein signaling such as Noggin [30]. However, the possibility of niche-independent growth of stem cells, associated with the accumulation of multiple mutations, cannot be excluded [31]. Genotype–phenotype analyses at a single-patient level could provide insights into adrenocortical tumorigenesis and patient-centered therapeutic development.

Moreover, we have analyzed the associations between the expression of CD90 and LGR5 with such clinico-morphological parameters as a histological subtype of ACC, Ki67 proliferative index, ENSAT stage, and survival status. Even in such a small sample, statistically significant differences in disease-free survival depending on the number of CD90-positive cells were found. It has been previously shown that increased CD90 expression is associated with worse overall and disease-free survival in other malignancies such as hepatocellular carcinoma, intrahepatic cholangiocarcinoma, gallbladder cancer, breast cancer, chondrosarcoma, gastric cancer [32,33]. Thus, our data are consistent with the results of studies on the prognostic role of CD90 in other organs. However, no significant differences in CD90 or LGR5 expression with other oncologically significant parameters were identified in the current study. It requires analysis of these CSC markers in a larger cohort of patients with ACC to reveal its potential diagnostic, prognostic, and predictive value.

## 5. Conclusions

The results of this pilot study demonstrate the presence of LGR5- and CD90-positive tumor cells in ACC, confirming the importance of studying CSC markers in both basic and clinical research. The identification and examination of CSC-specific markers in ACC offer a wide range of possibilities for targeted killing of these cell populations, potentially contributing to the cessation of tumor growth and prevention of disease recurrence.

## Figures and Tables

**Table 1:** Clinical and morphological characteristics of the studied sample of patients.

Sign	N	Me [Q1; Q3]
Age, years	13	54 [37; 59]
Tumor size, cm	13	8 [5; 9,5]
Ki-67 proliferative index, %	13	15 [10; 25]
Conventional subtype	3	20 [15; 60]
Oncocytic subtype	3	10 [5; 15]
Myxoid subtype	3	10 [7; 20]
Mixed (conventional + myxoid) subtype	4	27.5 [10; 60]
DFS, months	13	16 [2; 32]
OS, months	13	20 [16; 34]
**Sign**	**N**	**%**
T stage		
T1	4	30.8
T2	4	30.8
T3	4	30.8
T4	1	7.7
N stage		
N0	10	76.9
N1	3	23.1
M stage		
M0	11	85.0
M1	2	15.0
ENSAT		
I	4	30.8
II	4	30.8
III	3	23.0
IV	2	15.4
Hormonal activity of the tumor		
Non-functional	8	61.5
Hypercorticism	2	15.4
Hyperaldosteronism	1	7.7
Hypercorticism and hyperaldosteronism	2	15.4
Histological subtypes of ACC		
Conventional	3	23.0
Oncocytic	3	23.0
Myxoid	3	23.0
Mixed (conventional + myxoid)	4	30.8

Note: DFS—disease-free survival, OS—overall survival, ACC—adrenocortical cancer.

**Table 2:** Analysis of the number of CD90+ cells in different histological subtypes of ACC. Statistical analysis was undertaken with Kruskal–Wallis.

Sign	Conventional Subtype		Oncocytic Subtype		Myxoid Subtype		Mixed Subtype		*p*-Value
Number of CD90+ cells beneath the tumor capsule, cells/mm^2^	N	Values	N	Values	N	Values	N	Values	0.163
	3	91; 118; 21	3	18; 91; 139	3	6; 3; 10	4	57; 204; 4; 26	
Number of CD90+ cells in the tumor parenchyma, cells/mm^2^	N	Values	N	Values	N	Values	N	Values	0.161
	3	207; 230; 160	3	16; 0; 156	3	5; 13; 20	4	28; 621; 5; 320	

**Table 3:** Analysis of the LGR5 expression in different histological subtypes of ACC. Statistical analysis was undertaken with Kruskal–Wallis.

Sign	Conventional Subtype		Oncocytic Subtype		Myxoid Subtype		Mixed Subtype		*p*-Value
Expression of the LGR5 marker in scores	N	Values	N	Values	N	Values	N	Values	0.199
	3	3; 1; 2	3	2; 3; 2	3	1; 1; 1	4	2; 1; 1; 3	

## Data Availability

The data presented in this study are available in this article.
